# A role for the alpha-8 integrin chain (itga8) in glomerular homeostasis of the kidney

**DOI:** 10.1186/s40348-020-00105-5

**Published:** 2020-10-01

**Authors:** Ines Marek, Karl Friedrich Hilgers, Wolfgang Rascher, Joachim Woelfle, Andrea Hartner

**Affiliations:** 1 Department of Pediatrics and Adolescent Medicine, University Hospital of Erlangen, Loschgestrasse 15, 91054 Erlangen, Germany; 2 Department of Nephrology and Hypertension, University Hospital of Erlangen, Ulmenweg 18, 91054 Erlangen, Germany

**Keywords:** itga8, Glomerular homeostasis, Mesangial cells, Glomerulonephritis

## Abstract

Glomerulonephritis results in a dysregulation of glomerular cells and may end up in chronic alterations and subsequent loss of renal function. Therefore, understanding mechanisms, which contribute to maintain glomerular integrity, is a pivotal prerequisite for therapeutic interventions. The alpha-8 integrin chain seems to be an important player to maintain glomerular homeostasis by conferring mechanical stability and functional support for the renal capillary tuft.

## Introduction

Several forms of glomerulonephritis (e.g. IgA nephritis) are among the most common acquired renal diseases in childhood [1]. Although glomerulonephritis in many cases heals spontaneously, there is still a part of cases ending up in chronic renal disease with reduced kidney function. The mechanisms contributing to healing of the glomerular damage and preventing progressive loss of renal function are yet incompletely understood. Dysregulation of glomerular mesangial cells is frequently seen in various forms of glomerulonephritis [2], especially in mesangioproliferative IgA nephritis. Mesangial cells are one major cell type of the glomerulus, together with podocytes and endothelial cells of the capillary tuft. In other diseases, such as diabetic nephropathy or membranous glomerulopathy for instance, epithelial cells, especially podocytes, are primarily damaged. Endothelial cell diseases in turn are due to subendothelial immune complex deposition or the attachment of anti-glomerular basement membrane antibodies (Goodpasture syndrome) or due to an activation of coagulation (HUS) [3].

Mesangial cells not only offer a structural support for the capillary tuft, but also regulate capillary tone owing to their contractile properties. Furthermore, they serve as non-professional phagocytes to clear the capillary filtration apparatus from cellular debris [4]. Upon activation, mesangial cells are able to synthetize numerous cytokines and matrix molecules contributing to glomerulosclerotic changes [5]. Thus, mesangial cells are important players in preserving glomerular homeostasis.

## Integrins

Integrins are heterodimeric glycoproteins, consisting of an alpha and a beta subunit. The majority of these integrins functions as a receptor to extracellular matrix proteins via non-covalent binding. Depending on the combination of alpha and beta subunits, they form receptors for different ligands. Integrin receptors are pleiotropic, that is, one integrin can bind several ligands and one ligand can be recognized by several integrins [6, 7]. Ligand binding results in conformational changes of integrins to confer intracellular signalling cascades to influence cell shape by rearrangement of the cytoskeleton and to affect cell behaviour, such as cell differentiation, survival, proliferation and migration [8–10].

## The alpha-8 integrin (itga8) chain

itga8 is an integrin chain which is predominantly expressed on mesenchymal cells (vascular smooth muscle cells, some fibroblasts, mesangial cells). It forms a heterodimer with itgb1 to serve as a receptor for RGD-containing matrix molecules (fibronectin, vitronectin, tenascin C, osteopontin and nephronectin), similar to itgav and itga5 integrins [11]. itga8 is known to have important functions for kidney development. The critical developmental step of the kidney is the reciprocal interaction of the epithel of the ureteric bud (arising from the Wolffian duct) and its surrounding metanephric mesenchyme, which is necessary to induce ureteric bud outgrowth and differentiation of nephrons from the metanephric mesenchyme. Defects in these processes can result in congenital abnormalities of the kidney and urinary tract (CAKUT) [12]. Mice deficient for itga8 display reduced renal mass or even renal agenesis [13]. itga8 is normally expressed on cells of the condensing mesenchyme and ligates nephronectin (an extracellular matrix protein with an RGD motif, [14]), which in turn is expressed by epithelial cells of the ureteric bud. Under normal circumstances this interaction induces a signalling cascade to enhance expression of GDNF in the metanephric mesenchyme. In case of a deficiency of itga8, this epithelial-mesenchymal interaction is disturbed, and as a consequence, sprouting of the ureteric bud is impaired [15]. The resulting underexpression of GDNF finally results in a low nephron endowment [16]. Recent human genetic studies confirm a role of itga8 for kidney development, as mutations in the itga8 gene can result in renal agenesis in humans as well [17]. Expression of itga8 in the kidney is not confined to renal development, but can be detected in the differentiated kidney as well [18].

## itga8 in the glomerulus

**Fig. 1:**
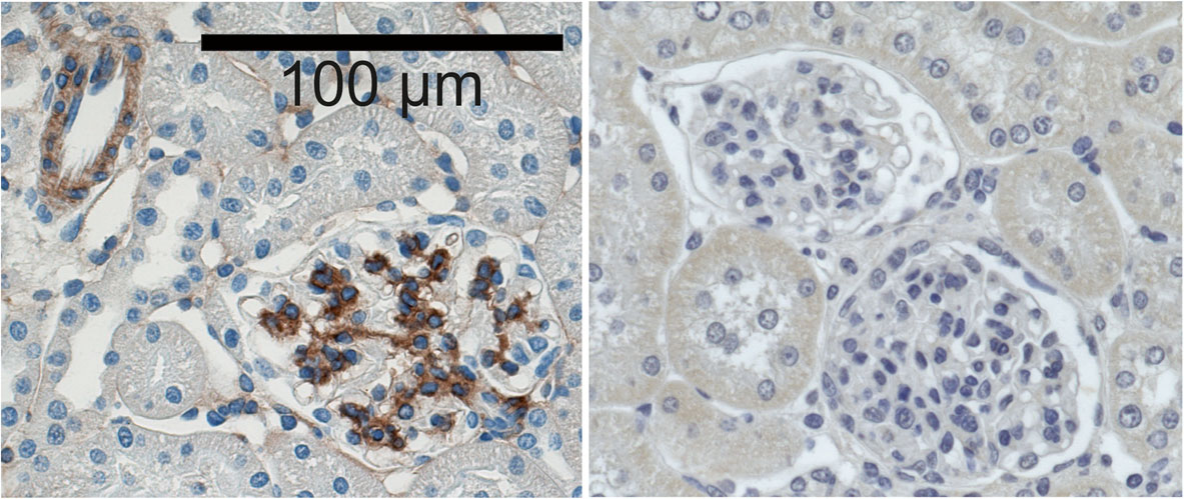
Left: Immunohistochemical detection of itga8 in rat renal tissue (brown staining). Strong reactivity is detected in the mesangium of the glomerulus and some reactivity is detected in vascular smooth muscle cells. Right: Negative control using diluted preimmune serum instead of primary antibody

**Fig. 2:**
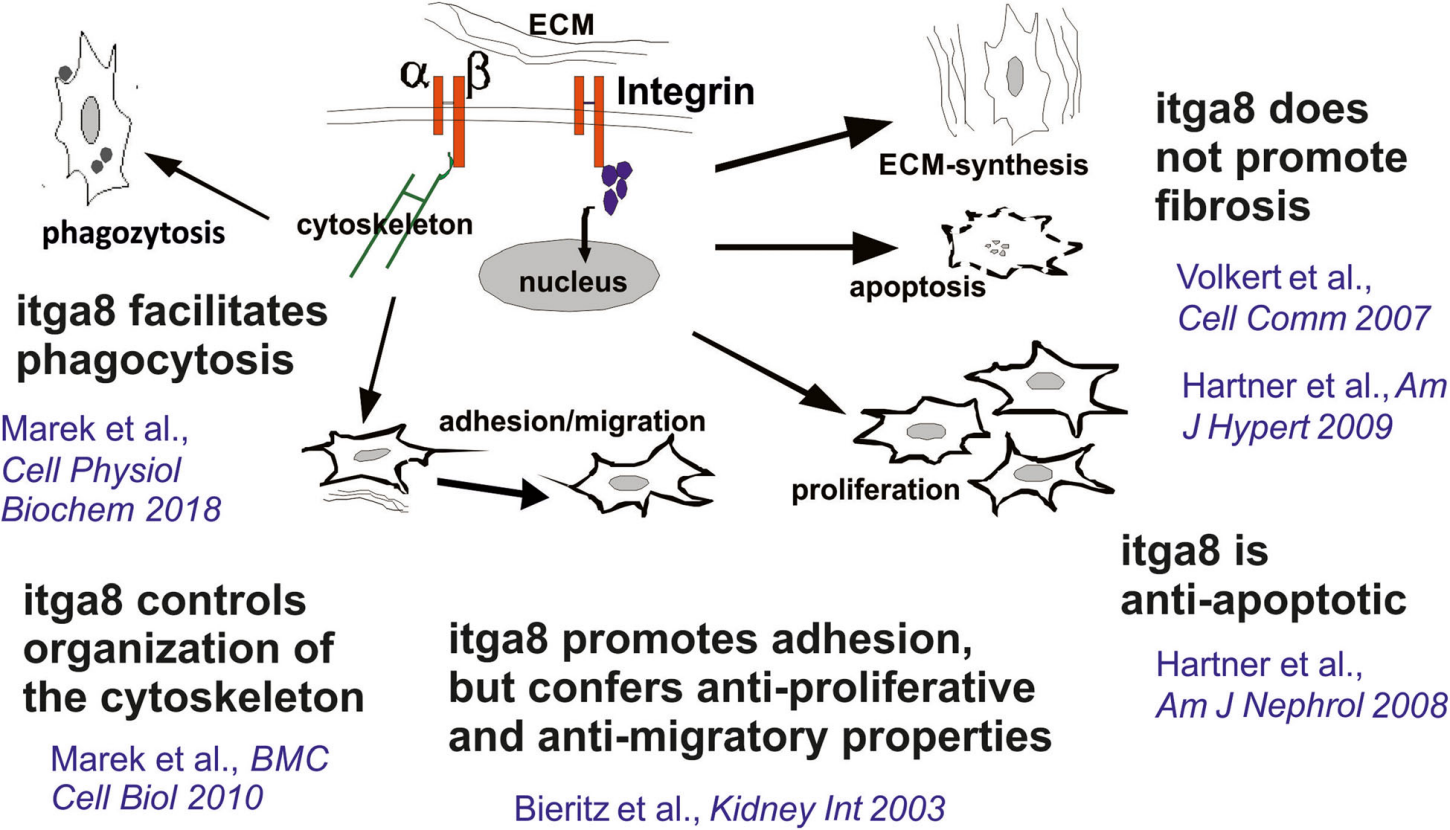
Schematic drawing of the functional properties of itga8 in mesangial cells

In the glomerulus, itga8 is specific for mesangial cells [18] (Fig. 1). This was recently confirmed by Lu et al. using single cell sequencing of mesangial cells [19]. There has been growing evidence for important regulatory properties of itga8 for the regulation of mesangial cell behaviour (see Fig. 2). In vitro studies using isolated wild type and itga8-deficient mesangial cells revealed that itga8 contributes to mesangial cell adhesion and spreading, while suppressing migration [20]. Therefore, itga8 might support firm adhesion of mesangial cells in vivo, conferring mechanical stability of the glomerulus. In accordance with this notion, it has been shown that itga8 increases mechanical stability of the glomerular capillary tuft in the DOCA-salt model of glomerular hypertension [21]. IgA nephropathy is a special form of glomerulonephritis characterized by hypercellularity as a consequence of mesangial proliferation [22]. Apoptosis of surplus cells and phagocytosis of extracellular matrix components and apoptotic cells are part of the clearing mechanisms and healing in glomerulonephritis [23, 24]. Suggestive of a role of itga8 for healing of glomerulonephritis, proliferation was increased in mesangial cells, which were deficient for itga8 [20], a feature that seems to be cell type specific, because a knock down of itga8 reduced proliferation in epithelial cells [25]. itga8 not only seems to attenuate proliferation in mesangial cells, but also appears to protect cells from apoptosis, resulting in a low cell turnover rate [26, 27]. In mice deficient for itga8, healing of glomerulonephritis is delayed as apoptosis remains increased for a longer time than in wild type mice [27]. Some integrins are suspected to promote the expression of matrix molecules and subsequent fibrosis [28]. This is not true for itga8, which did not act profibrotic, neither in vitro [29], nor in vivo [30]. In vitro studies suggested that itga8 supports the mesenchymal phenotype of cells, because lack of itga8 results in loss of actin stress fibres and reorganization of the cytoskeleton [31]. This may result in a reduced contractility of the cell. These findings are supported by data obtained from vascular smooth muscle cells, where itga8 expression promotes the contractile phenotype [32]. Finally, itga8 expression seems to increase the phagocytotic capacity of cells [33]. The most effective phagocytosis of apoptotic cells (leukocytes or mesangial cells) as well as of matrix components was detected in the presence of itga8 on mesangial cells.

## Conclusion

Taken together, there is strong evidence that itga8 supports essential functional properties of mesangial cells, like conferring mechanical stability, contractility and the ability to get rid of apoptotic cells and matrix debris from the glomerular filtration apparatus. itga8 is an important player in the proper function of the capillary tuft. itga8 signalling might aid in the resolution of glomerulonephritis and reduce the risk of chronification of glomerular disease. In view of the protective properties of itga8, targeting itga8, e.g. in an attempt to ameliorate fibrosis, does not seem reasonable to reduce renal injury. In contrast, expression of itga8 might serve as a risk stratification marker for the outcome of glomerular disease.

## Data Availability

Not applicable

## Abbreviations

DOCA: Desoxycorticosterone acetate
GDNF: Glial-derived neurotrophic factor
itg: Integrin chain
